# Peak Week Carbohydrate Manipulation Practices in Physique Athletes: A Narrative Review

**DOI:** 10.1186/s40798-024-00674-z

**Published:** 2024-01-13

**Authors:** Kai A. Homer, Matt R. Cross, Eric R. Helms

**Affiliations:** 1 Sport Performance Research Institute New Zealand (SPRINZ), Auckland University of Technology, 17 Antares Place, Rosedale, Auckland, 0632 New Zealand; 2 Department of Exercise Science and Health Promotion, Muscle Physiology Laboratory, Florida Atlantic University, Boca Raton, FL USA

**Keywords:** Physique sport, Bodybuilding, Carbohydrate, Muscle glycogen, Contest preparation, Peak week

## Abstract

**Background:**

Physique athletes are ranked by a panel of judges against the judging criteria of the corresponding division. To enhance on-stage presentation and performance, competitors in certain categories (i.e. bodybuilding and classic physique) achieve extreme muscle size and definition aided by implementing acute “peaking protocols” in the days before competition. Such practices can involve manipulating nutrition and training variables to increase intramuscular glycogen and water while minimising the thickness of the subcutaneous layer. Carbohydrate manipulation is a prevalent strategy utilised to plausibly induce muscle glycogen supercompensation and subsequently increase muscle size. The relationship between carbohydrate intake and muscle glycogen saturation was first examined in endurance event performance and similar strategies have been adopted by physique athletes despite the distinct physiological dissimilarities and aims between the sports.

**Objectives:**

The aim of this narrative review is to (1) critically examine and appraise the existing scientific literature relating to carbohydrate manipulation practices in physique athletes prior to competition; (2) identify research gaps and provide direction for future studies; and (3) provide broad practical applications based on the findings and physiological reasoning for coaches and competitors.

**Findings:**

The findings of this review indicate that carbohydrate manipulation practices are prevalent amongst physique athletes despite a paucity of experimental evidence demonstrating the efficacy of such strategies on physique performance. Competitors have also been observed to manipulate water and electrolytes in conjunction with carbohydrate predicated on speculative physiological mechanisms which may be detrimental for performance.

**Conclusions:**

Further experimental evidence which closely replicates the nutritional and training practices of physique athletes during peak week is required to make conclusions on the efficacy of carbohydrate manipulation strategies. Quasi-experimental designs may be a feasible alternative to randomised controlled trials to examine such strategies due to the difficulty in recruiting the population of interest. Finally, we recommend that coaches and competitors manipulate as few variables as possible, and experiment with different magnitudes of carbohydrate loads in advance of competition if implementing a peaking strategy.

## Key Points

- Physique athletes regularly implement “peak week” strategies based on the endurance training research.
- At present it appears that carbohydrate (CHO) loading strategies may increase muscle size; however, the effects on overall aesthetic performance are unknown.
- Due to a lack of data, it is difficult to make detailed peak week recommendations. Rather, it may be advisable to load with 3–12 g/kg/BM of CHO to increase muscle glycogen content, with this broad range representing different individual and divisional requirements. To optimise the magnitude of CHO load, coaches and competitors could establish an individual response pattern before competition by practicing and trialling peaking strategies in similar physiological conditions to peak week, and by using information from previous competitions. Further, manipulating as few variables at a time as possible could have the greatest physiological and psychological benefits.
- Experimental designs which assess visual physique changes while placing participants in ecologically valid physiological conditions are needed to fully elucidate the effects of CHO, water, and electrolyte manipulation peaking strategies.

## Background

In competition, physique athletes are subjectively judged and ranked on muscle size, proportion, symmetry, bodyfat levels, and posing ability on the day. Accordingly, stronger performers maximise these variables by implementing appropriate pre-competition nutrition and training strategies [1, 2]. In recent studies, contest preparation typically consists of at least four months of energy and thus carbohydrate (CHO) restriction in conjunction with increased training volumes [3–5]. The final week leading into competition is termed “peak week” and involves further manipulation of nutrition and training variables to improve appearance, ostensibly by increasing muscle glycogen (and thus muscle size) while minimising subcutaneous water (supposedly enhancing muscular definition) and abdominal bloating [3, 5, 6]. Feasibly, a greater understanding of how to manipulate core nutritional factors around peak week, notably CHO, could result in a more successful “peak” and improved performance.

**Fig. 1:**
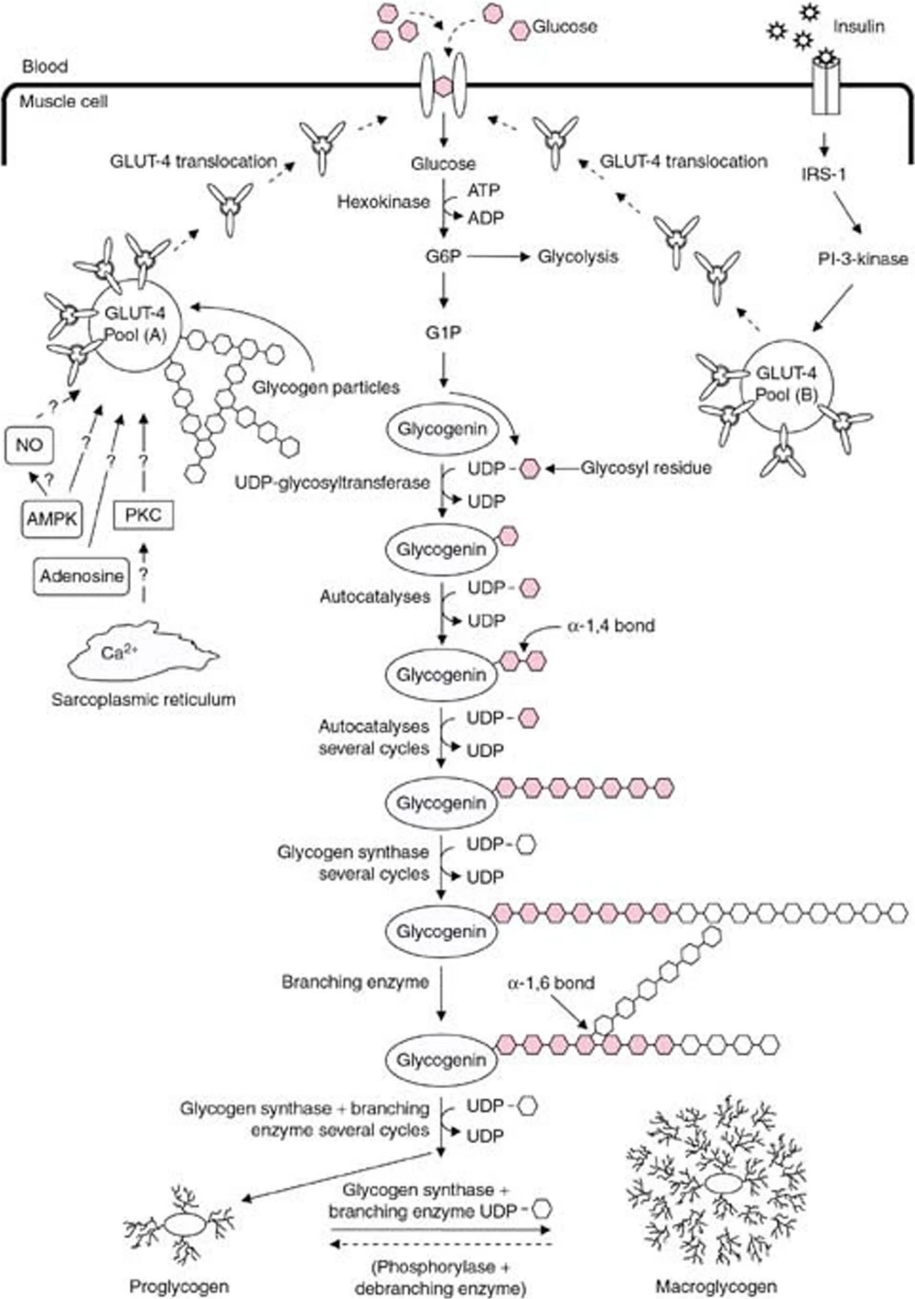
Schematic representation of the glycogenesis pathway in skeletal muscle. ADP = adenosine diphosphate; AMPK = adenosine monophosphate-activated protein kinase; ATP = adenosine triphosphate; Ca^2+^  = calcium ions; G1P = glucose-1-phosphate; G6P = glucose-6-phosphate; GLUT-4 = glucose transporter-4; IRS-1 = insulin receptor substrate-1; NO = nitric oxide; PI-3-kinase = phosphoinositide 3-kinase; PKC = protein kinase C; UDP = uridine diphosphate. From Jentjens and Jeukendrup [32]. The reader is also directed to the following reviews for further detail on the current understanding of the physiological processes and determinants of glycogenesis [8, 12, 33, 34]. Material is not part of the governing OA license but has been reproduced with permission. Determinants of Post-Exercise Glycogen Synthesis During Short-Term Recovery, Jentjens et al., Sports Medicine, 33, Springer Nature, 2012, reproduced with permission from SNCSC

Glycogen is the storage form of glucose derived from dietary CHO, of which skeletal muscle is the largest store within humans [7] (see Fig. 1 for a graphical representation of the glycogenesis pathway). Muscle glycogen is heterogeneously distributed between and organised in three distinct subcellular compartments (intramyofibrillar, intermyofibrillar, and subsarcolemmal spaces) within myofibers [8, 9]. The time course for full intramuscular saturation through supercompensation is variable and likely occurs 36–48 h following the cessation of the last exercise bout and CHO ingestion [10–12]. Amongst other factors, the rate of glycogenesis depends on total CHO and energy intake, sensitivity to and levels of serum insulin, prior glycogen depletion, muscle contraction-stimulated translocation of glucose transporters, gastrointestinal transport protein density, and relevant enzymatic activity [12–22]. Intramuscular glycogen size and density vary based on the subcellular site [23, 24] and total muscle glycogen content, with the larger macroglycogen particles stored with greater saturation two to three days into loading [25, 26]. Subcellular distribution is also dependent on training adaptations, where intermyofibrillar and subsarcolemmal glycogen content are greater in resistance-trained individuals than endurance-trained athletes [27, 28]. While CHO loading can increase muscle size through muscle glycogen content [29–31], the effect of individual glycogen particle volume and its subcellular distribution on muscle size and appearance is unknown. Feasibly, a better understanding of these physiological processes would allow physique athletes to adopt more specific nutritional and training strategies to enhance performance.

CHO loading protocols were first studied in endurance athletes, measuring performance and muscle glycogen levels, with muscle glycogen supercompensation observed following depletion and CHO loading [35–38]. Physique athletes subsequently adapted such strategies, manipulating CHO intake and training to enhance the appearance of muscle size [6]. However, muscle size changes in response to a CHO load are rarely an outcome measure in endurance training research, and the impact of loading on appearance is not relevant to endurance athletes. Muscle size increases in physique athletes have only been observed recently within a quasi-experimental design [30] and two case studies [39, 40], highlighting a paucity of empirical evidence to validate and guide these strategies. This review will highlight gaps in the literature, and subsequently provide suggestions for future research. Furthermore, relevant CHO loading trials are described while previously published information specifically relating to CHO manipulation strategies employed by physique athletes in peak week is examined.

## Carbohydrate Manipulation Practices in Endurance Athletes and Application to Physique Athletes

### Carbohydrate Loading Studies in the Endurance Literature

The study of interactions between muscle glycogen content, diet, and exercise performance began with a series of Swedish experimental trials in the 1960s utilising the then novel percutaneous muscle biopsy technique [35–38, 41–43]. The aim of this research was to investigate the effect of muscle glycogen as a stored energy substrate on endurance performance and the determinants of subsequent glycogenesis. While the effects of CHO loading on appearance lack relevance to endurance athletes, the findings of these trials have implications for physique athletes seeking to increase muscle glycogen content and enhance muscle size. Of the designs which manipulated diet, muscle glycogen supercompensation was observed from the consumption of a predominantly CHO diet following exhaustive, glycogen depleting exercise [35–38]. Further experimentation with large CHO loads scaled to bodyweight (ranging from 9 to 12 g/kg/day) for two to three consecutive days yielded significant muscle glycogen increases within the context of endurance training [44–49]. For example, McInerny et al. [47] depleted muscle glycogen content from 435 ± 57 to 96 ± 50 mmol/kg dry weight (DW) (*p* < 0.01) in the vastus lateralis of six well-trained endurance athletes with an exhaustive cycling protocol. Two days of CHO loading with 12 g/kg/day following the protocol resulted in supercompensation to 713 ± 60 mmol/kg DW (*p* < 0.01).

Similarly, Goforth et al. [49] implemented a three-day exercise and diet-induced (53 ± 9 g CHO/day) glycogen depleting protocol followed by a three-day repletion (720 ± 119 g CHO/day) without exercise in 14 male endurance athletes. Muscle glycogen content in the vastus lateralis increased from 408 ± 168 to 729 ± 222 mmol/kg DW (*p* ≤ 0.05). This supercompensated state was then maintained over the next two days with a moderate-CHO intake (332 ± 41 g). The preservation of muscle glycogen following supercompensation [49, 50] could be advantageous for physique athletes who prefer to load CHO earlier in the week, further away from competition. Indeed, this protocol is known as CHO “front-loading”, whereby competitors load at the start of peak week which theoretically allows more time to adjust nutritional intake according to appearance [5, 6].

In another study, Nygren et al. [31] leveraged magnetic resonance imaging to show vastus lateralis (+ 3.2%, *p* = 0.001) cross-sectional area and thigh circumference (+ 2.7%, *p* = 0.009) increases, coinciding with increased muscle glycogen content from 281 ± 42 to 634 ± 101 mmol/kg DW in five male participants. These changes were due to a four-day glycogen depleting protocol involving a low-CHO, high-fat diet with exhaustive exercise followed by four days of a high-CHO and low-fat diet without exercise. While promising, a small sample size and accordingly reduced statistical power constrains the generalisability of the results. Nonetheless, these findings indicate that intramuscular glycogen content changes may affect muscle size.

Hypothetically, glycogen-mediated muscle size increases are driven by increased intramuscular water as water molecules are bound to each stored glycogen particle [51–53]. The water bound to each particle is variable and seemingly determined by hydration status [53], although glycogenesis is likely not impaired by dehydration [54]. In a dehydrated state, Olsson and Saltin [52] concluded that at least three to four grams of water are stored intramuscularly with each gram of glycogen; however, changes in water content were measured at the whole-body level using tritium labelled water and not directly in muscle tissue.

Within a crossover trial that measured intramuscular water via muscle biopsy samples, Fernández-Elías et al. [53] created two experimental conditions where a CHO syrup was consumed with or without a rehydrating volume of water following cycling in the heat. Both groups consumed a CHO drink, with the rehydrating group consuming additional water to match individual fluid losses. Although both groups experienced similar glycogen repletion four hours following ingestion, muscle water content was higher in the rehydrating group than the non-rehydrating group (3814 ± 222 vs. 3459 ± 324 g/kg DW, *p* < 0.05), with 17 g of water bound to each gram of glycogen in the rehydrating group compared to only 3 g in the non-rehydrating group; accordingly, substantially increasing muscle volume via concurrent CHO and fluid ingestion may be relevant in the context of physique athletes. However, as muscle water content did not reach baseline levels in either group, strategies involving dehydration may not be advisable. It is also unknown if emphasising hydration status in physique athletes could impact the appearance and performance in other ways, as some authors hypothesise that higher levels of body water increase subcutaneous tissue thickness (ST), which may obscure muscular definition, while acknowledging that the efficacy of strategies to manipulate hydration status requires further examination [1].

### Dissimilarities Between Endurance and Physique Athletes

The theoretical underpinning and rationale for physique sport CHO loading protocols was born from endurance research. However, as endurance athletes are unconcerned with the aesthetic effects of CHO loading, research on the topic is not necessarily relevant or practical for physique athletes. Furthermore, the physiology of physique athletes at the end of contest preparation may be different from that of the typical endurance athlete. While some physique athletes potentially engage in high volumes of cardiovascular exercise [55–57], the prolonged periods of dieting, characterised by extreme reductions of both CHO and fat with the goal of achieving exceptionally low body fat, far below endurance athletes [39, 58–60] prior to CHO loading, differentiate the athletes. Additionally, physique athletes’ serum insulin concentrations decrease throughout contest preparation, considerably below the reference range in the week preceding competition [58, 59]. Given these physiological differences, it is difficult to directly apply literature-based endurance protocols to physique sport and doing so may not enhance aesthetic performance.

Unlike physique athletes during peak week, the goal of the endurance athlete is to fully saturate muscle and liver glycogen stores to reduce the likelihood of muscle glycogen depletion and hypoglycaemia, and their negative performance effects [34, 61, 62]. Endurance athletes likely have greater glycogenesis rate and capacity compared to physique athletes in peak week from their habituation to a high-CHO diet and the absence of extensive energy restriction. Thus, implementing endurance-based protocols in physique athletes may lead to greater CHO consumption than can be digested and absorbed in the gastrointestinal tract and synthesised as glycogen before competition [16, 19, 63–65]. This is especially relevant as physique athletes theorise that when CHO consumption exceeds total glycogen storage capacity and/or the maximal rate of glycogenesis, glucose accumulates in other body compartments, including the interstitial space of the subcutaneous layer [5], increasing compartmental fluid volume from the osmotic effect of glycogen on water [52]. This rise in subcutaneous water is thought to blur definition, an effect known as “spilling over” which detracts from muscle definition—often called “conditioning” in bodybuilding circles [1, 5]. Hence, the implementation of CHO loads of the same magnitude as utilised by endurance athletes may not translate to competitive success in physique sport.

### The Female Menstrual Cycle and Implications for Physique Athletes

**Fig. 2:**
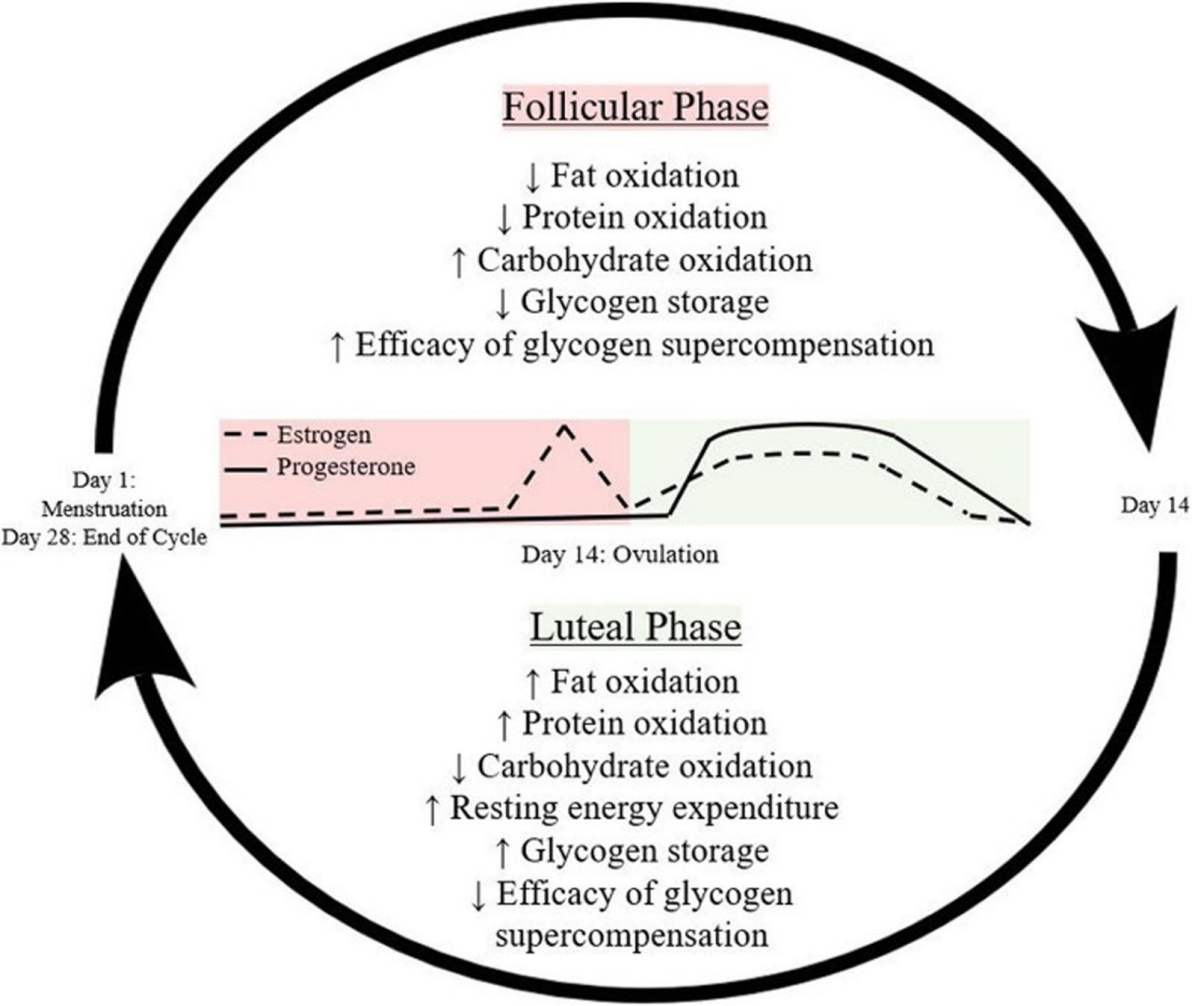
Schematic representation of key metabolic adaptations throughout the follicular and luteal phases of the menstrual cycle due to the physiological effects of oestrogen and progesterone. Image © Springer Nature from Wohlgemuth et al. [66] under Creative Commons Attribution 4.0 International License https://creativecommons.org/licenses/by/4.0/

In addition to the considerations described above, other physiological variables may be relevant. Notably, the effect of the menstrual cycle on glycogenesis following CHO loading in endurance athletes has been examined. For example, glycogen storage capacity decreases and the efficacy of supercompensation increases during the follicular phase, while the inverse occurs in the luteal phase [66] (see Fig. 2). Although the underlying mechanisms have yet to be fully understood, and a comprehensive examination is beyond the scope of this paper, menstrual phase-specific differences may be mediated by increased expression of oestrogens on glycogen synthase, insulin secretion, and adipocyte free-fatty acid oxidation [67–70]. Thus, muscle glycogen storage is theoretically elevated in the luteal phase compared to the early follicular phase [67]; however, large CHO loads have induced supercompensation to similar values in both menstrual phases in some trials [46, 71], but not in others [72, 73]. Given this ambiguity, it is difficult to make menstrual cycle phase-specific recommendations for CHO loading magnitudes for female competitors. Furthermore, female competitors commonly experience menstrual cycle disruption and hypothalamic amenorrhea close to competition due to low adiposity and energy availability from extreme dieting [74–80]. Chronic low energy availability reduces oestrogen and progesterone levels below-normal physiological ranges [81], which may impair muscle glycogen storage following a CHO loading protocol.

The theoretical variability in response to CHO intake during different phases of the menstrual cycle, or with hypothalamic amenorrhea, highlights the importance of individualised nutritional approaches to physique sport peaking. To better anticipate aesthetic changes and establish an individual response pattern, female competitors may benefit from experimenting with different CHO loads throughout the menstrual cycle during contest preparation (assuming it is present). Such an approach may provide information on CHO load magnitude and timing to inform future peaking strategies. Male competitors could also benefit from individualisation trial runs, potentially to a greater degree than their female counterparts, as their physiological response may be more consistent, although research is needed to confirm if relevant sex differences exist.

## Observational Designs in Physique Athletes

### Cross-Sectional Designs

While studies regarding CHO loading in physique competitors are likely more relevant than those concerning endurance performance, they are rare. Nevertheless, the few cross-sectional examinations that exist (summarised in Table 1) provide insight into peaking strategies employed by physique athletes. For example, in a recent survey of peaking strategies, Chappell and Simper [6] reported that 91% of a sample of 81 natural British bodybuilders (*M* = 59, *F* = 22) implemented some form of CHO manipulation. Of the peak week strategies included in the 34-item questionnaire, CHO manipulation was the most employed, where restriction was followed by loading in competitors who utilised both. Qualitative responses indicated that both restriction and loading phases lasted up to four days, with the aim of depleting muscle glycogen stores before inducing supercompensation to increase muscle size. Specific competition-day strategies were also recorded, with 71.6% consuming high-glycaemic index CHO 30–60 min prior to competition and 39.5% CHO loading. While surveying only a specific sample of physique athletes, these data indicate that CHO manipulation strategies are prevalent and popular.

**Table 1:** Summary of reviewed cross-sectional studies relating to peak week CHO manipulation in physique athletes

References	Sample	Methods	Relevant findings
Mitchell et al. [82]	Seven experienced (min. national level), natural M bodybuilders (36.7 ± 14.6yrs)	Subjective interviews regarding pre-competition training and nutrition, including “peak week”, were conducted by members of the research team	Six participants most recently utilised a modified CL strategy. Four participants had also previously implemented a traditional CL protocol which was described as not resulting in any discernible changes in appearance while inducing psychological distress
Chappell et al. [6]	Eighty-one (59 M, 22 F) competitive British bodybuilders (*M* = 33 ± 12, *F* = 34.7 ± 9.7yrs)	Distribution of a 34-item questionnaire regarding pre-competition training and nutrition to the combined 2016 and 2017 British Natural Bodybuilding Federation competitor dataset	Peak week CHO manipulation was the most prevalent strategy. Amongst competition-day strategies, high GI CHO pre-stage and higher CHO consumption were most prevalent

*CL* carbohydrate loading, *CHO* carbohydrate, *GI* glycaemic index, *M* male, *F* female

Similarly, albeit with a smaller sample, Mitchell et al. [82] interviewed seven experienced bodybuilders (10.4 ± 3.4 years’ experience and 14.3 ± 5.9 competitions) to elucidate their adopted contest preparation nutritional strategies and associated rationale. Six participants used a modified CHO loading regimen involving increasing CHO and concurrently tapering training. Specifically, one participant detailed having a higher intake (400 g) earlier in the week preceding two to three days of modest restriction (as low as 250 g) before increasing CHO to 300-400 g the day preceding competition. Four participants also reported implementing a CHO “backload”, which involved a three-day depletion followed by loading. Notably, there was dissatisfaction with the protocol, due to its perceived inability to induce appreciable changes in appearance and the psychological distress caused.

Experiences of psychological distress (i.e. increased tension, anxiety, anger, depression, and fatigue) are in line with studies of bodybuilders indicating prominent mood disturbances around the end of contest preparation [59, 74, 83]. Mood states likely degrade during contest preparation due to the extended period of energy restriction leading to low energy availability and the very low bodyfat levels achieved, far below competitors’ lower intervention point [84]. Mood disturbances could also be attributed to competition-day anxiety, potentially amplified by CHO loading prompting fears of “spilling over”. Researchers have proposed that psychological stress can negatively affect appearance through increased secretion of adrenocortical hormones, intensifying sodium reabsorption and potentially expanding extracellular fluid volumes [1, 85]; however, the effect of such water retention on appearance is unexplored. Thus, further investigation into the effects of CHO manipulation strategies on mood disturbances over the entirety of peak week and quantifiable physique changes is required to determine associations of mood states with physique sport performance.

### Single-Subject Designs

While long-term case studies examining bodybuilders pre- and post-competition have been published, few report peak week strategies or their possible effects [59, 78, 86]. A recent case study by Barakat et al. [40] is the most detailed examination of the effects of CHO manipulation on body composition outcomes to date; specifically, a natural male competitor followed a peak week protocol devised by the research group [1]. CHO consumption on the first day of data collection (nine days out from competition) was 297 g, which was reduced to 88, 73, and 88 g the preceding three days of depletion (six to four days out), respectively. CHO loading involved 582 g and 573 g the following two days (three to two days out), respectively, before tapering to 399 g the day before competition. The pattern of fat intake was inverse to CHO, where the highest intakes (86-132 g) occurred during CHO depletion. Finally, water intake also followed a somewhat similar pattern to CHO consumption from nine to two days out, with the lowest intake on the final day before competition. This was described as an attempt to reduce body water while preserving intramuscular glycogen and triglyceride stores with the cessation of physical activity.

Overall, there were favourable outcomes due to these combined strategies. The sum of ultrasound measures of muscle thickness (MT) collected from four sites (distal and proximal quadriceps, chest, and elbow flexors) was positively associated with CHO intake from the previous day throughout peak weak (*τ* = 0.733, *p* = 0.056). Prior to depletion, the sum of MT was 18.56 cm which increased to 18.99 cm the morning of competition. Relative quadriceps and chest MT increased, while elbow flexors decreased when comparing measurements from the previously mentioned data collection points. Indeed, total MT (+ 2.32%) and ST (− 0.67%) alterations were observed from the start of the protocol, as desired. With that said, it is challenging to untangle the individual effects of any single aspect of the combined peaking strategy within a case study design, which included manipulations of CHO, water, and dietary fat.

For instance, it is debatable whether CHO restriction is required to induce subsequent maximal glycogen supercompensation. Notably, equivalent and maximal muscle glycogen supercompensation can be achieved without prior cessation of dietary CHO [10, 11], which may indicate that depletion is not necessary, and leaves the question of whether comparable body composition changes could have been achieved with a more consistent CHO intake. Likewise, the strategy employed by Barakat et al. [40] of increasing fat intake while depleting CHO is known as “fat-loading” and is an attempt to increase intramuscular triglyceride content and thus muscle size. While no experimental evidence exists on fat-loading, this approach is rationalised by the comparable energy contents of intramuscular triglyceride being higher than glycogen [87]. However, as appreciable muscle size changes are likely driven by the water bound to glycogen rather than its energy density, the extent to which fat-loading increases muscle size may be negligible and the practice may simultaneously increase ST, as there is no known mechanism for preferentially storing triglycerides intramuscularly rather than subcutaneously.

Most importantly, it is difficult to determine the “visual” effects of this protocol on the participant’s physique, as there was no subjective judging or quantification of the competitor’s appearance. While anthropometric measurements indicated success, there are no data which correlate anthropometric changes with visual changes. Notably, the lack of visual, subjective assessments (e.g. photograph physique score changes on a 1–10 scale by a panel of qualified physique judges) is a persistent limitation of physique athlete case studies.

Another case study, conducted by Schoenfeld et al. [39], documented the effect of CHO loading on MT during contest preparation. In the final week before one of the participant’s four competitions, ultrasound MT was obtained at four sites (elbow flexors and extensors, midthigh and lateral thigh). Measurements were collected following a three-day depletion phase, the subsequent two-day loading phase, and finally one hour after the previous measure following CHO ingestion. The athlete decreased energy to 1474–1642 kcal/day and CHO to 20–46 g/day during depletion, lower than the lowest two-week rolling average intake during contest preparation (1953 kcal and 104 g/day), which was then increased during loading to 3374–3537 kcal/day and 449–483 g/day, for energy and CHO, respectively. The authors reported 5% and 2% upper arm and quadriceps MT increases, respectively, at the post-loading measurement compared to the post-depletion phase, and no changes following the post-loading 50 g CHO bolus. While MT increased after loading, the increases were observed post-depletion. However, the authors did not provide baseline MT data before depletion, whether the post-loading MT values improved upon pre-depletion values remain unknown. Thus, the efficacy of the strategy cannot be assessed since it is possible that similar final MT values could have been achieved without a peak week strategy. Future research should compare baseline outcome measures with post-depletion and loading values to better evaluate peaking strategies.

Additionally, further case studies provide indirect insight into the effects of CHO manipulation on body composition. For example, Rossow et al. [59] followed a white, male professional natural bodybuilder for 12 months pre- and post-competition. The authors reported increased body water (60.48–62.12L) and decreased body fat (6.6–4.5%) and sum of ultrasound ST (11 sites, 0.85–0.68 cm) a week before competition versus a month prior. These changes corresponded with the highest weekly mean energy intake and a marked blood glucose increase from three months prior (52–72 mg/dL). While total CHO intake was unreported, the increased energy intake, body water, and blood glucose may be attributed to increased CHO as part of a peaking strategy. Similarly, Halliday et al. [75] reported a modest increase in mean CHO intake to 3.8 g/kg in the final week of a female figure competitor’s contest preparation from 3.4 and 2.7 g/kg at weeks one and 10, respectively. Energy intake was also the highest recorded since week three of contest preparation, corresponding with a skinfold thickness reduction from four weeks prior. However, as CHO intake was reported as weekly means and not as specific daily intakes, it is difficult to discern if a specific peaking protocol was implemented. Despite indications of potential CHO manipulation in both Rossow et al. [59] and Halliday et al. [75], it is difficult to interpret which specific protocols were implemented and their potential efficacy.

While a unique nutritional intake during “peak week” which includes CHO manipulation itself is a popular strategy amongst physique athletes [6], the specific pattern and magnitude of CHO can vary widely. For example, Steen et al. [88] documented the use of a traditional CHO loading regimen by a drug-enhanced male bodybuilder. The competitor restricted CHO for three days before loading with 300 g the day before and on competition day. Likewise, Hickson et al. [89] also detailed the use of a similar protocol by another enhanced male bodybuilder, who depleted CHO for two days before loading with only 100 g for the next three days before competition. Contrastingly, a very high intake of CHO was captured within a clinical case report of a professional bodybuilder admitted to intensive care due to bilateral lower limb paralysis [90]. The athlete reported consuming minimal CHO in the month preceding competition before loading with 800 g of high-glycaemic index CHO on competition day. While no anthropometric data were collected in these case studies [88–90], they highlight substantial variability in peak week approaches. All relevant case studies are summarised in Table 2.

**Table 2:** Summary of reviewed case studies relating to peak week CHO manipulation in physique athletes

References	Participant	Methods	Relevant findings
Hickson et al. [89]	White, drug-enhanced M amateur bodybuilder (27yrs)	Collection of daily food records over the course of a 30-day contest preparation period	Implementation of a 2-day CHO restriction period followed by 3 days of moderate intake in the week before competition. Consumed mainly CHOs prior to stepping on stage. Placed in top three of competing weight class, no body composition outcomes measured
Steen et al. [88]	White, drug-enhanced M amateur bodybuilder (25yrs)	Collection of five-day food records at 6 and 5 months, and 1 week pre-competition	Implementation of 3-day CHO restriction, followed by 3 days of moderate intake in the week before competition. No body composition outcomes measured
Rossow et al. [59]	White, natural M bodybuilder (26yrs)	Monthly body composition assessment (via 4CM and SFs) and collection of dietary records for 6 months pre- and post-competition. On the month of competition, data were collected 1 week pre-competition	↑ FFM and TBW with ↓ ΣUST and BF% (both 4CM and SFs) in month of competition compared to previous month
Halliday et al. [75]	White, natural F amateur figure competitor (26yrs)	Collection of SF thickness measures on eight and four occasions pre- and post-competition, respectively. Daily self-reported dietary intake and BM	↓ ΣSF thickness in the final week before competition from four weeks prior, coinciding with a modest ↑ in energy and CHO intake
Lapinskienė et al. [90]	Natural M professional bodybuilder (28yrs)	Case report of a competitor admitted to hospital following bilateral lower limb paralysis	The competitor consumed 800 g of high GI CHO on the day of competition following a month of severe CHO restriction. The exact amount of CHO consumed pre-stage is unknown
Schoenfeld et al. [39]	White, M amateur bodybuilder (25yrs)	Monthly body composition over an 8-month pre-competition and 4-month post-competition period with daily nutrition logs completed by the participant. The peaking strategy for one of the participant’s four competitions was detailed	↑ in UMT observed following 3-day CD and 2-day CL phases
Barakat et al. [40]	Middle Eastern-American, natural M professional bodybuilder (29yrs)	Collection of dietary intakes, hydration status, and body composition of the participant on 6 days over an 8 day “peaking” period	A 3-day CD followed by a 2-day CL and 1-day tapering phases implemented prior to competition. UMT positively correlated with CHO intake. ICW/ECW peaked on the final day of CD, ↓ during CL and ↑ to slightly above baseline value on competition day

CHO = carbohydrate, 4CM = four compartment model, SF = skinfold, ↑ = increased, FFM = fat-free mass, BM = body mass, TBW = total body water, ↓ = decreased, Σ = summation, UST = ultrasound subcutaneous tissue thickness, GI = glycaemic index, CD = carbohydrate depletion, CL = carbohydrate loading, UMT = ultrasound muscle thickness, ICW/ECW = intracellular water to extracellular water ratio

### Multiple-Subject Designs

While the physique sport literature predominantly consists of case studies, there are some multiple-subject studies which may provide more generalisable findings (see Table 3 for a summary of multiple-subject observational studies). Bamman et al. [29] followed six male bodybuilders for twelve weeks preceding competition. Unfortunately, despite stating a CHO load commenced 72 h before competition and reporting a mean CHO intake (290 ± 73 g) from a three-day dietary profile completed the same day as the commencement of loading, day-to-day dietary intake was undisclosed. In the final 24–48 h preceding competition during CHO loading, ultrasound biceps MT reportedly increased (+ 4.9%), while the ST measure from the same site had decreased (− 29.4%) from six weeks prior; however, the results should be interpreted with caution, since neither met the threshold for statistical significance (*p* > 0.05). Further, due to the unclear results, the time between data collection and the lack of detailed day-to-day nutritional information, direct causal inferences cannot be drawn from this study.

**Table 3:** Summary of reviewed group-level observational studies relating to peak week CHO manipulation in physique athletes

References	Sample	Methods	Relevant findings
Lamar-Hildebrand et al. [92]	Ten F (six bodybuilders, four non-competitors)	3-Day food records and self-report questionnaires on weeks 8, 4, and 2 prior to and the weekend of competition	Competitors increased total energy and CHO intake on the week and weekend of competition. No placing or body composition data reported
Bamman et al. [29]	Six enhanced M bodybuilders (25-29yrs)	Collection of 3-day food records and biceps UMT and UST on weeks 12, 6 and 0 pre-competition	All competitors engaged in a CL protocol 72 h before competition. From weeks 6 to 0 before competition, UMT↑ while UST↓ (both non-significant)
Walberg-Rankin et al. [91]	Six F bodybuilders (27.3 ± 5.1yrs)	Collection of food records from 28 to 26, 9 to 7, and 2 to 1 day(s) pre-competition, competition day to 2 days post, and 19–21 days post-competition	↑CHO and ↓fat consumption in the 2 days prior to competition in comparison to 9–7 days pre-competition
Nunes et al. [95]	Eleven untested M state-level bodybuilding and physique competitors (28.8 ± 4.1yrs)	Body composition assessment of competitors in the afternoon the day before and on competition day. Relevant outcome measures include muscle girths and BW fractions derived from single-frequency BIA. No dietary intakes recorded	No changes in girths and significant ↑ in ICW, ICW/ECW, and TBW were observed. Hypothesised by authors that this was induced by CHO manipulation

CHO = carbohydrate, UMT = ultrasound muscle thickness, UST = ultrasound subcutaneous tissue thickness, CL = carbohydrate loading, ↑ = increased, ↓ = decreased BIA = bioelectrical impedance analysis, ICW = intracellular water, ICW/ECW = intracellular water to extracellular water ratio, TBW = total body water

In two studies which assessed dietary intakes but did not track body composition changes of female bodybuilders, CHO intake increased in the immediate days prior to competition [91, 92]. Walberg-Rankin et al. [93] reported increased CHO consumption two days before competition compared to data collected one and three weeks prior. Specifically, this involved an almost twofold group-level CHO intake increase (202.7–385.9 g, *p* = 0.001), accounting for 83% of total energy. Similarly, Lamar-Hildebrand et al. [92] drew comparisons between in-season and off-season bodybuilders and made similar observations. The competitors increased energy intake (1283 ± 789 to 2228 ± 1192 kcal) on the weekend of competition, driven by higher CHO consumption (222 ± 149 to 359 ± 194 g). While these group-level observational studies demonstrate the use of CHO loading strategies amongst female bodybuilders and their magnitudes, the efficacy of these practices cannot be determined due to the absence of body composition data. To summarise, both case study and multiple-subject observational studies indicate that CHO manipulation is a common strategy amongst physique athletes; however, the positive impact on anthropometry hinted at by this literature remains an untested assumption.

In addition to CHO manipulation, physique athletes may concurrently manipulate electrolyte and water intake when peaking [94]. This practice is intended to increase intracellular water (ICW) while decreasing extracellular water (ECW), supposedly to expand muscle and reduce subcutaneous water, respectively [1, 40, 95]. This theory is rationalised by the high concentration of sodium and potassium in ECW and ICW, respectively, associated with cell fluid volume (i.e. the sodium potassium pump) [96]. Consequently, bodybuilders and researchers propose that increasing potassium while reducing sodium intake alters cellular concentrations of these ions, which when combined with increased muscle glycogen content, creates an osmotic gradient for interstitial water to be drawn into muscle [1, 40, 95]. The proposed outcome of such process is a favourable ICW/ECW ratio, which may enhance the appearance of muscle fullness and definition [1]. As such, techniques to estimate the distribution of fluid compartments in the context of peaking are of interest to the physique sport population [40, 95].

To examine if such fluid shifts are indeed achieved by bodybuilders via peaking protocols, researchers have adopted bioelectrical impedance analysis (BIA) and bioelectrical impedance spectroscopy (BIS). For example, Nunes et al. [95] employed a single-frequency BIA device to compare competition-day body water fraction changes from the day prior in 11 male competitors. Each participant achieved simultaneous ICW increases and ECW decreases, increasing their ICW/ECW ratio as presumably intended. While the lack of dietary data is a limitation, the authors hypothesised the bodybuilders manipulated CHO, electrolytes, and water, causing these outcomes. While promising, methodological limitations complicate these findings [95]. In particular, hydration status, diet, and acute water intake were, understandably, uncontrolled. Unfortunately, single-frequency BIA results are sensitive to and impacted by these variables [97]. Additionally, and most importantly, single-frequency BIA cannot distinguish between intracellular and extracellular fluid compartments, as multiple frequencies, from devices such as multi-frequency BIA or BIS, are required to do so [98]. Thus, a prediction equation developed by Matias et al. [99] was utilised by Nunes et al. [95]; unfortunately, since the equation was derived from high-level non-physique athletes, disparities in the body geometries between the sample used for calibration and physique athletes probably inflated the already unacceptably high expected fluid compartment error estimations (± 3.6–6 kg of fluid). Further, the testing conducted by Matias et al. [99] to develop the equation was highly standardised, whereby participants were required to have been fasted for 12 h, be euhydrated, and not have exercised in the past 15 h, which likely differed from the testing conditions of Nunes et al. [95]. These methodological shortcomings and error rates confound interpretation, and likely account for the highly homogenous competition-day ICW/ECW ratios (1.92 ± 0.01L) reported by Nunes et al. [95], while also highlighting the difficulty of standardising BIA measurements of physique athletes during peak week.

Compared to the BIS-derived raw bioimpedance results from the aforementioned case study by Barakat et al. [40], a smaller competition-day ICW/ECW ratio (+ 3.87%) increase was reported from the day prior in comparison to Nunes et al. (+ 20%) [95], likely due to the different devices employed. BIS devices possess superior predictive capabilities compared to BIA as they use a spectrum of frequencies to differentiate ICW and ECW [98, 100], making the use of regression-derived population-specific prediction equations to estimate fluid compartments unnecessary [98, 101]. However, limitations still exist even within BIS. Specifically, device validation in different populations is required, as inherent body geometry and composition variations exist [98]. This limitation was present in Barakat et al. [40], as the extreme body geometry and composition of the participant likely diverged from the assumptions of the BIS device’s in-built equations.

Notwithstanding this limitation, an increased competition-day ICW/ECW ratio from the day prior was also reported by Barkat et al. [40] who also examined the effects of their peaking strategy on fluid compartment shifts. Curiously, however, the highest reported ICW/ECW ratio was three days prior to competition, the morning after the depletion phase when MT was at its lowest and ST at its second highest. Given the relationship proposed by Escalante et al. [1], Barakat et al. [40], and Nunes et al. [95] that a high ICW/ECW ratio should coincide with the best combination of MT increases and ST decreases, and therefore best appearance, it is plausible that either the proposed relationship is incorrect or that bioelectrical impedance derived ICW and ECW may not accurately represent body water changes during peak week.

Indeed, regarding this proposed relationship, attempting to induce such fluid shifts with the restriction of water and sodium while loading potassium—as commonly practiced by physique athletes—could even degrade aesthetic performance. Dietary sodium reductions may slow small intestine glucose absorption due to its down-regulating effect on the concentration of brush border GLUTs [16–18], while also reducing the concentration of sodium ions required for SGLT1 cotransport of glucose [102–105]. Additionally, SGLT1 and GLUT5 density and activity are lowered with a CHO-free diet [103]. While these adaptations begin within four hours of CHO exposure [106], it may take several days for appreciable increases in SGLT1 expression to occur [107], potentially slowing glucose absorption when initially loading CHO following depletion and sodium restriction. Furthermore, blood pressure decreases during the final weeks before competition [59], which would likely be compounded by sodium restriction [108]. Such blood pressure reductions would be disadvantageous for competitors seeking transient muscle size and definition increases from active hyperaemia and the accumulation of metabolites following a pre-stage “pump-up” routine [5, 109, 110]. Thus, it may even be advisable to increase sodium consumption on competition day for certain divisions due to its acute effect on raising plasma volume and blood pressure (albeit requiring further research to confirm the efficacy of this strategy) [5, 111–113].

This strategy is often justified by the misconception that ICW and ECW are equivalent to intramuscular and subcutaneous water, respectively, and that by increasing ICW via glycogenesis, water restriction will preferentially lead to higher proportional ECW decreases [1]; however, including water restriction as part of a peaking strategy may be deleterious for competitors. While intracellular fluid is indeed the major skeletal muscle fraction, it is also comprised of a non-negligible amount of extracellular fluid [114, 115]. Skeletal muscle is approximately 70–75% fluid [116, 117], and total muscle water content is reduced during dehydration [118, 119], potentially affecting muscle size. Intravascular plasma is also extracellular fluid [114, 120, 121]; thus, blood volume reductions from water restriction may impair the delivery of glucose to myocytes and therefore the efficacy of CHO loading. While the osmotic effect of glucose induces acute water shifts within these compartments [122], water balance and the concentration of ions are tightly regulated by homeostatic mechanisms [123, 124]. It has been proposed that the temporal lag in re-establishing homeostasis following water loading could be leveraged to increase urine output and therefore water excretion during subsequent restriction to reduce ECW, where increased intramuscular glycogen from CHO loading may preserve or increase muscle water and thus size [1]. However, there was a moderate relationship between TBW and ECW (*r* =  − 0.44. *p* < 0.05) in physique competitors with varied approaches to water intake during peak week as recently observed by Escalante et al. [125]. This indicates that the proportion of ECW is greater when TBW is reduced, which suggests that the competitors were not able to preferentially reduce ECW through peaking strategies. As the appearance of the participants was not subjectively evaluated, in addition to a lack of experimental evidence, the combined effect of water and electrolyte manipulation on the appearance of muscle and its time course is unknown. Furthermore, Escalante et al. [1] recommended that water and CHO manipulations be planned and practiced before peak week, or to be kept relatively constant if such practice runs are not feasible, highlighting the potential for performance decrements with such strategies.

Notably, a cross-sectional study examining the diets and metabolic profiles of male and female high-level drug-enhanced bodybuilders found that blood sodium levels were within normal ranges 24 h prior to competition [126]. This was despite the deliberate restriction of dietary sodium, evidenced by strategies such as the deliberate shift from tap water to distilled, to reduce fluid retention. As such, it seems unlikely that electrolyte and water manipulation substantially alter the concentration of sodium ions to induce the desired fluid shifts. In fact, if successful, such practices may increase the risk of life-threatening conditions such as hyperkalaemia and rhabdomyolysis, especially when combined with diuretics and anabolic steroids [127, 128]. Based on the physiological reasoning provided and the previously discussed studies not observing competitor appearance changes [40, 95], it is difficult to assert that such fluid shifts and the nutritional strategies intended to induce them occur as expected or are favourable for physique sport performance.

In summary, while observational studies document the implementation of CHO manipulation protocols by physique athletes and suggest that these techniques may increase muscle size, limited study numbers and methodological concerns confound interpretation. Furthermore, we present our arguments against certain strategies (such as water and electrolyte manipulation) which are predicated on physiological mechanisms rather than empirical evidence. Such proposed strategies may indeed improve appearance; however, to determine if that is the case requires rigorous and controlled investigations.

## Experimental Designs

### A Quasi-Experimental Design in Physique Athletes

Arguably the most relevant study of peak week was conducted by de Moraes et al. [30]. The researchers stratified 24 male bodybuilders into two groups, delineated by whether CHO was loaded or not before competition. Notably, MT appeared to increase following a 24-h CHO load after three days of depletion. Both groups increased daily CHO intake following depletion, with the loading group increasing to 9.0 ± 0.7 g/kg BM from 1.1 ± 0.4 g/kg BM compared to the non-loading group increasing to 5.2 ± 0.9 g/kg BM from 0.9 ± 0.6 g/kg BM. The loading group increased both elbow flexor (+ 3.1%, *p* < 0.05) and triceps brachii (+ 3.4%, *p* < 0.05) MT, whereas there were no increases within the non-loading group. The loading group also improved their physique silhouette scores on a scale developed by Castro et al. [129]. The competitors were evaluated using the silhouette scale by seven official bodybuilding judges blinded to the intervention, indicating that CHO loading may positively influence subjective measures of muscle size. However, a limitation of the silhouette scoring system employed is that any changes in the appearance of leanness may not be distinguished or quantified. Furthermore, skinfold measures were not collected at the second point of data collection, meaning the effect on ST could also not be determined. For future research, assigning a score for both muscle size and definition when subjectively evaluating the appearance of competitors may allow for further detail on the effects of peaking strategies to be uncovered.

Measures of abdominal and epigastric symptoms were also collected and compared between groups [30]. Constipation was the most prominent gastrointestinal symptom in both groups following depletion, which persisted within the non-loading group at the second point of data collection (2.00 ± 0.67 to 2.13 ± 0.81, *p* > 0.05). Contrastingly, incidences of constipation decreased in the loading group (1.89 ± 0.57 to 1.53 ± 0.72, *p* < 0.05) while diarrhoea increased (1.22 ± 0.42 to 1.93 ± 0.37, *p* < 0.05). This is potentially the result of drastically increasing CHO beyond the emptying rates of the stomach and gastrointestinal tract [16], where glucose transporters are seemingly downregulated following CHO restriction [130]. Interestingly, both groups’ total scores of gastrointestinal symptoms increased (loading group = 14.9 ± 0.22 to 16.93 ± 0.24, *p* < 0.05 vs. non-loading group = 13.88 ± 0.28 to 14.21 ± 0.31, *p* < 0.05). This finding may be indicative of competition stress, irrespective of CHO intake, since acute stressors can slow gastric emptying rates [131]. Thus, competition stress may contribute to the slowing of gastrointestinal glucose absorption and subsequent glycogenesis, as well as to gastrointestinal distress. The findings of de Moraes et al. [30] further highlight the utility of experimenting with different CHO loads prior to competition, as individualising the CHO loading protocol (i.e. the timing, quantity, and type of CHO) could maximise the rate of glycogenesis while minimising gastrointestinal symptoms. Such experimentation may confer some physiological and psychological benefits [132–134] associated with intermittent dieting or “refeeding”, while allowing for competitors to become (re)accustomed to large volumes of CHO.

### An Experimental Design

In the only experimental design to date, Balon et al. [135] intended to replicate a CHO loading protocol employed by bodybuilders with a crossover design. In conclusion, no significant muscle girth increases were reported following a two-day CHO loading regimen. The protocol involved a three-day isoenergetic, low-CHO diet (10% of diet) followed by an isoenergetic, high-CHO diet (80% of diet) for days during the experimental arm, while the control arm participants consumed an isoenergetic, moderate-CHO diet (55% of diet).

Unfortunately, this study did not replicate the peak week conditions of bodybuilders. Notably, the mean body fat percentage of the participants was 10 ± 1%, which is much higher than the values of 4.4–6.3% typical of high-level male bodybuilders in the final week of competition [39, 40, 80, 126]. The participants also had not dieted with a reduced CHO intake for months prior to the study. This detail is salient as contest preparation may induce chronic glycogen depletion which could subsequently impair glycogenesis. Further, the participants consumed an isoenergetic diet during depletion, whereas CHO loading physique athletes are initially in a severe energy deficit which would cause greater glycogen depletion prior to loading [3]. The participants also altered the proportion of CHO rather than increasing their energy intake with additional CHO, which may not have maximised glycogenesis [12, 15].

Furthermore, a high-volume resistance training protocol of 30–35 sets to or very close to failure was performed daily during depletion, which may vary from typical practices of bodybuilders (~ 50% higher than that used by natural bodybuilders [56]) who often decrease training stress during peak week [82, 88, 92]. Such high set volume and intensity during CHO restriction may have caused muscle damage and sarcolemmal membrane disruption, possibly impairing glycogenesis in the subsequent CHO load [136–140]. Indeed, it may be advisable to not train with high volumes, in close proximity to failure, as well as not performing exercises which train muscles at long lengths under heavy eccentric loads [136–143] during peak week to avoid excessive muscle damage.

Finally, while the authors did not report muscle girth increases, it is plausible that a visual change in the appearance of the muscle and overall aesthetic could have occurred. Therefore, further ecologically valid experimental research examining visual changes by judges of the relevant physique sport division with body composition measures is required to determine the effects of peaking strategies on bodybuilding performance. Both experimental designs are summarised in Table 4.

**Table 4:** Summary of reviewed experimental studies relating to peak week CHO manipulation in physique athletes

References	Sample	Study design	Methods	Relevant findings
Balon et al. [135]	Nine resistance-trained M (23 ± 4.4yrs)	Crossover	Experimental arm: 3 days of isoenergetic, low-CHO diet and intense resistance training followed by two days of isoenergetic, high-CHO diet and tapered resistance training Control arm: 3 days of isoenergetic, moderate-CHO diet and intense resistance training followed by two days of isoenergetic, moderate-CHO diet and tapered resistance training	No significant changes in muscle girths between groups
de Moraes et al. [30]	24 (15 CL, 9 NCL) South American, untested, M bodybuilders (CL = 27.3 ± 5.0, NCL = 26.2 ± 4.9yrs)	Quasi-experimental	Stratification of competitors into CL or NCL groups. Collection of UMTs, circumferences, gastrointestinal symptoms, and mood states following 3-day CD and 1-day CL. Photographs of the participants at data collection were rated by seven federated bodybuilding according to a photo silhouette scale [129]. Food diaries of the final 4 days pre-competition were collected	Both groups ↑ energy and CHO intake from the CD period for which the magnitude was greater in the loading group. ↑ in UMT, circumferences, and silhouette scores were observed in the CL group. Mood disturbances ↑ slightly in both groups, as did gastrointestinal symptoms which were greater in the CL group

CHO = carbohydrate, CL = carbohydrate loading, NCL = non-carbohydrate loading, UMT = ultrasound muscle thickness, CD = carbohydrate depletion, ↑ = increased

## Practical Applications

Based on the current evidence, making specific peaking recommendations to improve physique sport performance is difficult. Nevertheless, some practical guidance to prospective athletes and coaches wishing to adopt peaking strategies can be provided. For example, loading with 3-12 g/kg/BM of CHO may increase muscle size; however, the exact amount likely is dependent on the requirements of the individual and division of competition (i.e. male bodybuilders likely require more CHO than bikini competitors due to a greater emphasis on muscularity). Thus, it is likely advisable that competitors and coaches test different CHO loading magnitudes and strategies well in advance of competition day in comparable physiological conditions (i.e. very low levels of adiposity, typically one to two months away from competition). Visual changes and the time course for the CHO load to “take effect” and alter the competitor’s physique as well as the quantity and type of CHO consumed should be recorded to inform future peak week strategies to increase their reliability. Such practice runs also present competitors the opportunity to habituate to high acute CHO intakes and reduce gastrointestinal stress [12, 16]. Additionally, using information from previous competitions to guide future practice is recommended. Establishing an individual response pattern could be especially valuable for female competitors whose rate of glycogenesis and glycogen storage capacity may be impacted by disruptions to the menses typically seen in contest preparation [74–80]. Thus, it would be prudent for coaches and competitors to experiment with differing loads during different phases of the menstrual cycle (or in its absence) prior to competition to better anticipate visual changes.

During peak week, avoiding strategies that drastically alter nutritional variables from previous weeks may be sensible. These alterations, which include the substantial manipulation of CHO, water and electrolytes, and the introduction of new foods, could introduce the risk of unpredictable and deleterious effects if not executed appropriately. For example, loading with too much CHO may reduce the appearance of muscle definition. Additionally, depleting glycogen prior to loading may be unnecessary to achieve maximal glycogen supercompensation [10, 11], and thus, competitors can avoid extremely low-CHO intakes during peak week which may incur unnecessary psychological stress and reduce training quality [30, 59, 74, 144]. However, without experimental data to confirm our suppositions, it is possible that this approach could be advantageous in some cases (i.e. a competitor requiring lower body fat benefiting from low energy intake during depletion). Likewise, restricting water and sodium have the potential to reduce muscle size and vascularity, and impair CHO loading, while overconsumption may lead to unwanted water retention which may obscure muscle definition and/or cause abdominal distension [1].

As physique competitors typically incur psychological distress close to competition [30, 59, 74] and given the proposed relationship between stress and water retention [1], stress management may be an overlooked area to improve performance. Thus, to minimise stress, establishing an individual response pattern and reducing the number of variables manipulated may benefit the competitor. Psychological distress may also be amplified by travel-related stressors, whereby competitors could travel earlier and become accustomed to the new environment and time zone (if applicable) to lessen the impact on performance. Mindfulness techniques which have been shown to moderately reduce stress in non-clinical populations (Hedges’ *g* = 0.55, *p* < 0.01) [145] may also be of interest to competitors; however, further research examining such techniques in the context of contest preparation and peak week is required to make concrete recommendations.

The manipulation of training variables should be considered when attempting to induce muscle glycogen supercompensation. As glycogenesis may be impaired by high degrees of muscle damage, training with high volumes, very close to failure, or performing exercises which place muscles at long lengths or under heavy eccentric loads should be avoided [136–143]. It is also advisable that competitors consume adequate energy predominantly from high-glycaemic index CHO with minimal fibre to maximise glycogenesis while minimising gastrointestinal distress [16, 146]. Finally, as muscle glycogen levels remain stable for up to five days following supercompensation even with the cessation of exercise [49, 50], ceasing resistance training and cardiovascular exercise during and after loading may help maximise and preserve intramuscular glycogen for competition.

As some divisions emphasise muscularity of certain muscle groups (i.e. upper body for physique and lower body for bikini competitors), preferential supercompensation of glycogen may be achieved in these muscle groups if they are depleted to a greater degree via resistance training. As the rate of glycogenesis is influenced by prior glycogen depletion and muscle contraction-stimulated translocation of glucose transporters [12, 20–22], preferentially depleting muscle groups of interest may benefit certain competitors; however, further evidence is required to determine the effects on physique sport performance.

If feasible, it may be ideal for competitors to achieve the required level of conditioning three to four weeks prior to competition and slowly increase CHO intake. Such an approach might improve resistance training performance [144] while allowing time to adjust intakes based on physique changes (i.e. increasing CHO as much as possible without increasing ST). This approach may preclude the necessity of “last minute” or otherwise harmful, drastic nutritional changes such as dehydration or sodium restriction with potassium supplementation. Contrarily, consuming a concentrated bolus of sodium immediately prior to competition in conjunction with a pump-up routine may acutely enhance appearance in relevant divisions; nevertheless, this approach is speculative (as are many assertions in this area about best practice) and requires specific study.

## Conclusions

Despite the extent of its effect on physique performance being largely unexplored, CHO manipulation strategies are widely employed by physique athletes [6]. Only one quasi-experimental trial, one limited experimental trial, and few observational studies have examined CHO loading in physique athletes—highlighting a need for further, well designed studies of the topic. Accordingly, experimental designs which closely mimic the nutritional and training practices of bodybuilders and the physiological conditions they are in during peak week will help both practitioners and athletes implement appropriate peaking strategies to maximise physique sport performance. Notably, ideal peaking protocols may differ by many factors that are not yet well-explored in the literature, including competitor division as well as specific performance enhancing drug-use (or lack thereof). As recruitment of physique competitors is understandably difficult [95], further quasi-experimental designs comparing more diverse samples of physique athletes who utilise different strategies may be a feasible alternative to elucidate the interactions of these variables on physique sport performance.

## Data Availability

Not applicable.

## Abbreviations

CHO: Carbohydrate
DW: Dry weight
MT: Muscle thickness
ST: Subcutaneous tissue thickness
ICW: Intracellular water
ECW: Extracellular water
BIA: Bioelectrical impedance analysis
BIS: Bioelectrical impedance spectroscopy
SGLT1: Sodium–glucose cotransporter one
GLUT: Glucose transporter
